# *GlyT2*-Dependent Preservation of MECP2-Expression in Inhibitory Neurons Improves Early Respiratory Symptoms but Does Not Rescue Survival in a Mouse Model of Rett Syndrome

**DOI:** 10.3389/fphys.2016.00385

**Published:** 2016-09-12

**Authors:** Swen Hülsmann, Guillaume Mesuret, Julia Dannenberg, Mauricio Arnoldt, Marcus Niebert

**Affiliations:** ^1^Clinic for Anesthesiology, University Medical Center Göttingen, Germany; ^2^Center for Nanoscale Microscopy and Molecular Physiology of the Brain Göttingen, Germany; ^3^Institute of Neuro- and Sensory Physiology, University Medical Center Göttingen Göttingen, Germany

**Keywords:** neurodevelopmental disorders, autism spectrum, inhibition

## Abstract

Mutations in methyl-CpG-binding protein 2 (MECP2) gene have been shown to manifest in a neurodevelopmental disorder that is called Rett syndrome. A typical problem that occurs during development is a disturbance of breathing. To address the role of inhibitory neurons, we generated a mouse line that restores MECP2 in inhibitory neurons in the brainstem by crossbreeding a mouse line that expresses the Cre-recombinase (Cre) in inhibitory neurons under the control of the glycine transporter 2 (*GlyT2, slc6a5*) promotor (*GlyT2*-Cre) with a mouse line that has a floxed-stop mutation of the *Mecp2* gene (*Mecp2*^stop/y^). Unrestrained whole-body-plethysmography at postnatal day P60 revealed a low respiratory rate and prolonged respiratory pauses in *Mecp2*^stop/y^ mice. In contrast, *GlyT2*-Cre positive *Mecp2*^stop/y^ mice (Cre^+^*; Mecp2*^stop/y^) showed greatly improved respiration and were indistinguishable from wild type littermates. These data support the concept that alterations in inhibitory neurons are important for the development of the respiratory phenotype in Rett syndrome.

## Introduction

The neurodevelopmental disorder Rett syndrome (OMIM 312750, Rett, 1966) is caused by mutations in the gene for the methyl-CpG-binding protein 2 (MECP2). Although *MECP2* is a X-chromosomal gene, Rett syndrome usually describes the phenotype of girls with a heterozygous mutation that occurs at a prevalence of 1/10,000–1/15,000 female births. Rett syndrome is often inherited from maternal and paternal *de novo* germline mutations (Thomas, 1996; Girard et al., 2001) but also can be transmitted through generations if the X-chromosomal inactivation of the maternal mutation is skewed (Hoffbuhr et al., 2001). After a latent period of approximately 1–3 years (Rett, 1966) girls develop a neurological phenotype that includes stereotypic hand movements, seizures, and typically also autonomic dysfunctions with breathing abnormalities as well as mental retardation with loss of language skills (Jellinger, 2003). The clinical spectrum in boys with *MECP2*-mutations is more diverse, ranging from a classic form of the Rett syndrome (Masuyama et al., 2005) to severe cases of neonatal encephalopathy (Villard, 2007; Reichow et al., 2015).

Alterations of breathing are observed both in male and female patients with *MECP2*-mutations. The respiratory phenotype of female Rett syndrome patients is characterized by periods of hyperventilation alternating with prolonged periods of breath-holdings causing intermittent hypoxia (Julu et al., 2001), which often resemble apneustic breathing (Kerr and Julu, 1999). These disturbances of breathing are discussed as a main cause of sudden death (Kerr et al., 1997). In contrast, the male phenotype often exhibits respiratory insufficiency soon after birth with hypoventilation and prolonged apneas (Geerdink et al., 2002; Kankirawatana et al., 2006; Schüle et al., 2008).

There are many different mouse models available that resemble symptoms of the classical Rett syndrome (Ricceri et al., 2008). The male *Mecp2*^−/*y*^ null mouse is often used as a model (Guy et al., 2001) since it shows impaired locomotion including hind limb clasping and autonomic dysfunctions with respiratory abnormalities. However, it becomes evident that the typical alteration of breathing in male MECP2-deficient mice is rather hypoventilation that presents with a reduced respiratory rate and minute ventilation together with a high number of apneas (Viemari et al., 2005; Chao et al., 2010; Vogelgesang, 2013; Wegener et al., 2014), but not apneustic breathing (Stettner et al., 2007). These observations suggest that male MECP2-deficient mice might be a good model for breathing phenotype of male patients.

Nevertheless, both data from male MECP2-deficient mice and heterozygous female mice point toward alterations in inhibitory neurons that are involved in disease progression. For example, a knockout of the *Mecp2* gene in inhibitory neurons, using a VIAAT-specific expression of the Cre-recombinase, has been shown to be sufficient for the generation of respiratory phenotype (Chao et al., 1997). Although VIAAT is known to be expressed both in GABAergic as well as in glycinergic neurons (Chaudhry et al., 1998), the phenotype of the VIAAT-Cre induced *Mecp2*-KO was attributed to the loss of MECP2 in GABAergic neurons (Chao et al., 1997). Early changes of inhibitory synaptic transmission that are observed in *Mecp2*^−/*y*^ mice at P7 were also effecting the GABAergic system (Medrihan et al., 2008). Since GABA/glycine co-transmission is common at P7 in the respiratory system (Rahman et al., 2013) and VIAAT-levels in *Mecp2*^−/*y*^ mice were also reduced (Medrihan et al., 2008), we assume that alteration of synaptic inhibition is one of the key failures during early development. We therefore can hypothesize that breathing can be improved if MECP2 is introduced inhibitory neurons of the respiratory network.

The current experiments were designed to test if the breathing and/or the developmental progression of the disease is significantly improved by preventing the loss of MECP2 in inhibitory neurons of the brainstem and spinal cord, which are involved in the regulation of breathing (Richter and Smith, 2014). To achieve this goal we used a mouse line allowing expression of the Cre-recombinase in inhibitory neurons under control of the *GlyT2*-promotor (Ishihara et al., 2010; Rahman et al., 2015) that was crossbred to a global knock-out of *Mecp2*, which has been achieved by a knock-in of a loxP-site flanked stop codon into the locus of the *Mecp2* gene (*Mecp2*^Stop/y^ mice; Guy et al., 2007). We focused on changes of breathing, that are typically observed in the male mouse models of the Rett syndrome, regardless whether *Mecp2* is knocked out in all cells (Guy et al., 2001) or only in inhibitory neurons (Chao et al., 2010).

## Methods

### Animals and genotyping

Animals were bred in the central animal facility of the University medical center of the Georg-August University Göttingen and treated in accordance with the German Protection of Animals Act (TierSchG) and with the guidelines for the welfare of experimental animals issued by the European Communities Council Directive 2010/63/EU. The file number of the Lower Saxony authorities (Nds. Landesamt für Verbraucherschutz und Lebensmittelsicherheit, LAVES) is 33.9-42502-04-15/1764. Heterozygous female founder mice (B6.129P2-*Mecp2*^tm2Bird^/J; *Mecp2*^stop/x^) were ordered from The Jackson Laboratory (Stock Number: 006849). *GlyT2*-Cre (Tg(*Slc6a5*-iCre)^121Veul^) mice were originally provided by Volker Eulenburg (Erlangen). Mating was always made from *Mecp2*^stop/x^ heterozygous female (Guy et al., 2007) and *GlyT2*-Cre positive male mice (Ishihara et al., 2010). Both lines were maintained on a C57/B6 background. After birth all offspring were raised by foster mice.

Genotyping was performed using standard PCR technique described in the original publication of the *GlyT2*-Cre mice (Ishihara et al., 2010) or as provided by The Jackson Laboratory (https://www2.jax.org/protocolsdb/f?p=116:1:0::NO:::) for the *Mecp2*^stop/x^ mice.

### Immunohistochemistry

Mice were deeply anesthetized using isoflurane (1-Chloro-2,2,2-trifluoroethyl-difluoromethylether, Abbott, Germany) and perfused transcardially using 4% paraformaldehyde (PFA) in phosphate buffered saline (PBS). Brains were removed and post-fixed in 4% PFA in PBS for 24 h and stored in PBS until used for sectioning. Before sectioning, brains were equilibrated in HEPES buffer (7.5 g NaCl, 0.3 g KCl, 0.06 g KH_2_PO_4_, 0.13 g Na_2_HPO_4_, 2 g Glucose, 2.4 ml 10 mM HEPES, 0.1 g MgCl_2_, 0.05 g MgSO_4_, 0.165 g CaCl_2_, pH 7.4) for 48 h, cryoprotected in 15% sucrose in PBS for 24 h followed by equilibration in 30% sucrose in PBS for 24 h at 4°C, and then frozen at −80°C. Series of 30-μm-thick brain sections ranging from cervical spinal cord to midbrain colliculi were cut using a freezing microtome (Frigocut, Reichert-Jung). Sections were stored in HEPES buffer. All buffers were supplemented with small amount sodium azide (NaN_3_). Antigen retrieval was performed in citrate buffer (10 mM citric acid, 0.05% Tween20, pH 6.0) at 80°C for 30 min. Sections were incubated in blocking buffer (PBS, 0.1% Triton-X100, 1% Tryptone/Peptone) for 60 min at RT to permeabilize and block non-specific binding. Primary antibody (rabbit anti-MECP2, Cell signaling, cat. No. 3456S) was diluted 1:400 in blocking buffer and incubated for 60 min at RT. After rinsing in buffer (PBS, 0.05% Tween20, 0.3% Triton X100), sections were incubated for 1 h at RT in the dark with anti-rabbit atto647-conjugated secondary antibodies (Sigma-Aldrich, Cat. No. 40839) diluted 1: 400 in blocking buffer. During washing, sections were counterstained with DAPI and mounted onto microscope-slides and coverslipped with fluorescent mounting medium (DAKO). Immunofluorescence was analyzed with an Axio Imager.Z1 fluorescence microscope (Zeiss) equipped with a mercury vapor short-arc lamp (HBO100, Zeiss), filters for DAPI (Ex. 335–383 nm, Bs. 395 nm, Em. 420–470 nm) and Cy5 (Ex. 625–655 nm, Bs. 660 nm, Em. 665–715 nm) and a digital camera (AxioCam MRm, Zeiss). Images were taken at 10x magnification with Zeiss Zen2 software and imported into FIJI/ImageJ, digitally adjusted if necessary for brightness and contrast, and assembled into plates using CorelDraw Software. Composite pictures were generated by the Microsoft image composite Editor (ICE) software after background rolling ball subtraction (FIJI/ImageJ).

### Unrestrained whole-body-plethysmography

To determine parameters of ventilation we used unrestrained whole-body-plethysmography. Mice were placed in an acrylic glass chamber that was connected to a differential low-pressure TRD5700 Pressure Transducer (Buxco). The second channel of the pressure transducer was connected to a reference chamber that was connected to room air through a fine-mesh screen. Warming of the inspired air and cooling during expiration results in pressure changes (Drorbaugh and Fenn, 1955; Bartlett and Tenney, 1970). Since the chamber allows air to pass in and out through a defined resistor (a fine-mesh screen) the pressure changes in the chamber represent air flow (principle of a screen pneumotach). The leak in the chamber introduced a time constant for the decay of pressure changes of 40 ms. A negative pressure bias flow of 1 l/min was introduced by a small animal bias flow generator (DSI). The bias flow was also used to calibrate the chamber. Data were acquired using a PC-computer running Ponemah software (DSI). Flow signals were exported to ASCII and imported to LabChart software (ADInstruments), band-pass filtered (0.5–20 Hz) offline. Mice were allowed to explore the chamber for 12 min to adapt to the new environment before the measurement was started. For analysis of respiratory cycle length (inspiratory peak to peak interval) a period of 3 min was analyzed by the peak analysis module of LabChat software. Breathing frequencies were calculated as the reciprocal of the averaged inspiratory interval. Peak to peak intervals that were longer than 1 s (0.75 s) were counted during the 3 min period and were referred to as *number of apneas* >*1 s/3 min (*>*0.75 s/3 min)*. The coefficient of variation (CV) of the cycle period (inspiratory interval) and the irregularity score (IS = 100 ^*^ ABS[(Int_n_ − Int_n−1_)/Int_n−1_]) of the interval were calculated to assess variability of breathing (Wegener et al., 2014).

When the mice were transferred from the plethysmography chamber back to the cage, they were grasped by the tail and kept in air for up to 20 s to test for hind limb clasping. Hind limbs clasping (HLC) was noted if both hind limbs were retracted toward the abdomen. The numeric values (shown in Table 1) are % of animals showing HLC. The operator was not blinded to the genotype.

**Table 1:** **Summary of detected changes in ***Mecp2***-KO and ***GlyT2***-MECP2**.

	**WT**	**CTRL**	***Mecp2*-KO**	***GlyT2*-MECP2**
**GENOTYPE**				
*Mecp2*	**+/y**	**+/y**	***stop*** **/y**	***stop*** **/y**
*GlyT2*-Cre (BAC)	**neg**.	**pos**.	**neg**.	**pos**.
**AGE (P40;** < P55)	41.3 ± 3.2	40.9 ± 1.9	43.1 ± 4.7	41.4 ± 2.6
Number	10	19	9	14
BW (g)^A^	19.1 ± 2.7	20.4 ± 1.4	16.2 ± 2.3^#^	18.0 ± 2.9
HLC [%]^χ2^	0	0	16.7	0
BPM^A^	405.9 ± 39.8	372.2 ± 78.9	354.2 ± 81.0	320.6 ± 80.0
Apnea (>1 s)/3 min^AR^	0.4 ± 0.8	0.5 ± 1.3	7.0 ± 11.3^#^	2.0 ± 2.1
Apnea (>750 ms)/3 min^AR^	1.5 ± 1.6	1.5 ± 3.0	12.6 ± 14.5^*^^,^^#^	5.1 ± 4.6
CV cycle period^AR^	0.54 ± 0.11	0.50 ± 0.09	0.78 ± 0.27^#^	0.58 ± 0.19
IrrScore cycle period^AR^	0.34 ± 0.06	0.33 ± 0.06	0.48 ± 0.12^*^^,^^#^	0.36 ± 0.14
**AGE (P60;** P55-P70)	62.8 ± 4.4	63.6 ± 3.1	61.7 ± 3.0	60.6 ± 2.3
Number	9	16	11	10
BW (g)^AR^	24.0 ± 2.3	26.1 ± 1.8	21.3 ± 6.1^#^	24.4 ± 1.4
HLC [%]^χ^2^^	0	0	44.4^*^±^#^	0
BPM^A^	388.6 ± 54.7	394.4 ± 60.8	292.5 ± 104.7^*^^,^^#^	372.8 ± 68.0
Apnea (>1 s)/3 min^AR^	1.2 ± 2.4	0.3 ± 0.8	6.7 ± 6.8^#^	0.9 ± 1.5
Apnea (>750 ms)/3 min^AR^	2.7 ± 4.8	2.0 ± 2.3	14.1 ± 16.1^*^^,^^#^	3.2 ± 4.0
CV cycle period^A^	0.53 ± 0.15	0.51 ± 0.09	0.64 ± 0.16	0.57 ± 0.10
IrrScore cycle period^AR^	0.33 ± 0.06	0.33 ± 0.05	0.39 ± 0.11	0.37 ± 0.06

*Significant differences (p < 0.05 from the multiple comparison procedures) are indicated with asterisks (compared to WT) or octothorpes (compared to CTRL). Hind limb clasping (HLC) is given as the % of animals showing this symptom. Coefficient of variation (CV) and irregularity score (IrrScore) were calculated from the cycle periods. The type of test that was performed is indicated in the first column: ^χ2^Chi-square; ^A^ANOVA; ^AR^ANOVA on ranks. Data are given as mean ± standard deviation (SD)*.

### Statistical analysis

All tests were performed on a Microsoft Windows 10 PC using SigmaPlot (version 12.5; Systat Software GmbH). Analysis of variance (one way ANOVA) with all pairwise multiple comparison procedures (Holm–Sidak method) was used. ANOVA on ranks (Kruskal–Wallis) was used with an all pairwise multiple comparison procedures (Dunn's method), if the normality test (Shapiro–Wilk) failed. Motor phenotype was compared using a Chi-square test. The Kaplan–Meier estimator was used for survival analysis. For statistical comparison of survival, the Gehan–Breslowl test was used. Data are presented as mean ± standard deviation (SD).

## Results

### Immunostaining for MECP2 expression in hindbrain

**Figure 1:**
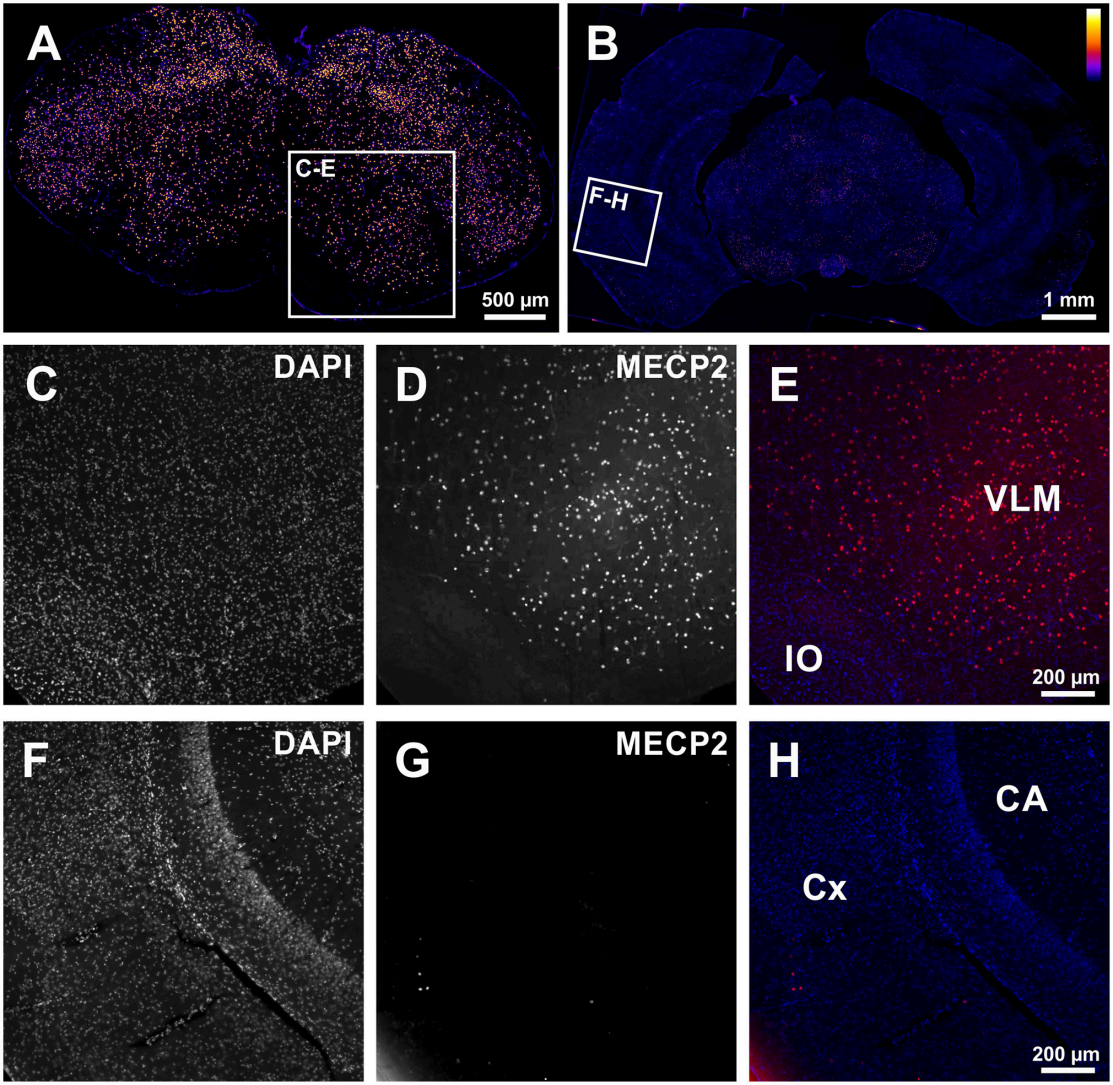
**(A,B)** Low magnification tiled images from the medulla **(A)** and forebrain **(B)** showing successful rescue of MECP2-expression in the brainstem of *Mecp2*^Stop/y^ mice using GlyT2-Cre in the brainstem of a Cre^+^; *Mecp2*^Stop/y^ mouse. **(C–E)** Image detail from **(A)** confirming the expression of MECP2 in the ventrolateral medulla (VLM). IO: inferior olive. **(F–H)** In the Cortex (CX) and Hippocampus (CA) only spurious expression of MECP2 is observed **(G)**. In panel **(E,H)**, DAPI staining is shown in blue, and MECP2/atto647 fluorescence is shown in red.

In a first set of experiments we tested if excision of the stop-cassette in *Mecp2*^stop/y^ mice by the expression of Cre-recombinase using *GlyT2*-Cre mice was effective to allow expression of MECP2 in hindbrain neurons. As shown in Figure 1, MECP2 expression was preserved in a subset of brainstem neurons of *Mecp2*^stop/y^ *GlyT2*-Cre positive mice. Notably only a few neurons expressed MECP2 in the cortex and hippocampus.

### Respiratory phenotype is diminished if MECP2-Expression is preserved in GlyT2-Cre mice

Mice were functionally analyzed in two groups (Table 1). The first group was tested at ~6 week (P40), when *Mecp2*^stop/y^ mice were reported to develop first symptoms (Guy et al., 2007). At this earlier stage we did not observe any difference in the respiratory rate nor in the number of apneas, that are a typical problem of the phenotype (Wegener et al., 2014). Also no significant change in the motor system was observed and only one *Mecp2*^stop/y^ mouse at this age showed hind limb clasping (Table 1). At P40 the breathing frequency of freely moving WT mice was 405.9 ± 39.8 breaths per minute (BPM; mean ± SD) in control (Cre^+^; *Mecp2*^+/*y*^) mice 372.2 ± 78.9 BPM was recorded. At this early stage, *Mecp2*^stop/y^ mice were indistinguishable from control mice. They had a breathing rate of 354.2 ± 81.0 BPM (*P* = 0.050), while in mice, in which the stop-codon was removed in inhibitory neurons (Cre^+^; *Mecp2*^stop/y^), the rate was 320.6 ± 80.0 BPM.

**Figure 2:**
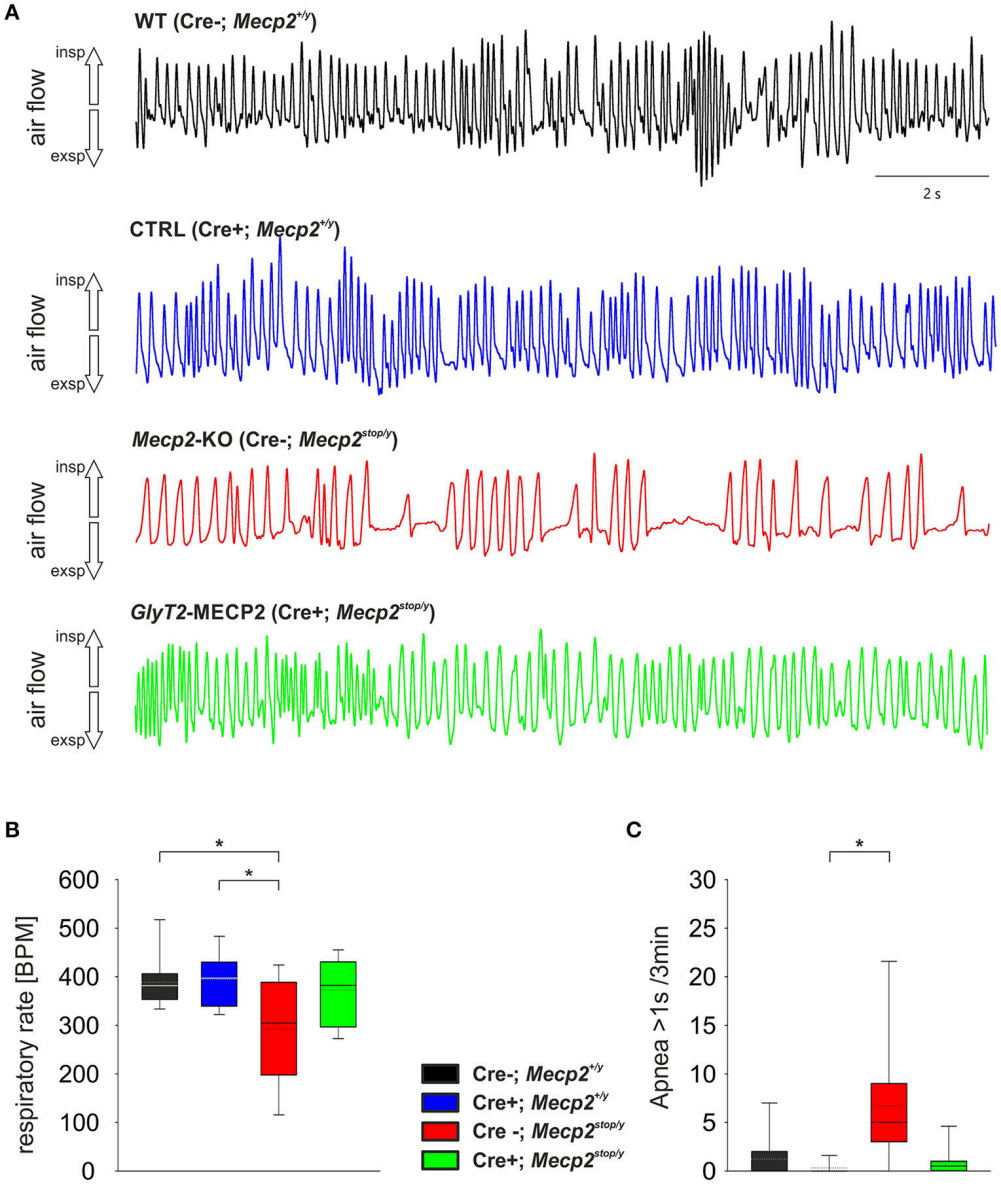
**(A)** Unrestrained whole-body plethysmography recordings from WT (black), Control mice (*GlyT2*-Cre; blue), MECP2-deficient *Mecp2*^stop/y^ mice (red) and mice, in which MECP2 was expressed in *GlyT2*-Cre positive neurons (*Mecp2*^stop/y^, Cre^+^; green). Trace show arbitrary unit (a.u.) of the flow. Inspiration is upward, expiratory flow is downward going. **(B,C)** Statistical analysis of the respiratory rate in breaths per minute (BPM; **B**) and the number of apneas that were larger than 1 s **(C)**. In the box plots the boundary of the box closest to zero indicates the 25th percentile, the solid lines within the box marks the median (the dotted lines are mean), and the boundary of the box farthest from zero indicates the 75th percentile. Whiskers (error bars) above and below the box indicate the 90th and 10th percentiles. Asterisks represent statistical significance (*p* < 0.05).

At P60 a significant change in the breathing was observed in *Mecp2*^stop/y^ mice (Figure 2). While WT mice (388.7 ± 54.7 BPM) and control mice (394.4 ± 60.8 BPM) showed a regular breathing, *Mecp2*^stop/y^ mice had only 292.5 ± 104.7 BPM (*p* = 0.030 vs. WT mice, *p* = 0.006 vs. control mice). In contrast, the breathing rate of mice in which the MECP2 was restored in hindbrain inhibitory neurons (cre^+^; *Mecp2*^stop/y^) was indistinguishable from WT and control littermates (372.8 ± 68.0 BPM; *p* = 0.873 vs. WT and *p* = 0.851 vs. control mice). A significant number of pauses that were longer than 1 s was only observed in *Mecp2*^stop/y^ *GlyT2*-Cre negative mice (Figure 2; Table 1). Although the motor phenotype was not completely developed at P60, hind limb clasping was observed only in 44.4% of *Mecp2*^stop/y^ mice but neither in control and WT mice nor *GlyT2*-Cre positive *Mecp2*^stop/y^ mice (Table 1).

### Survival is not improved

**Figure 3:**
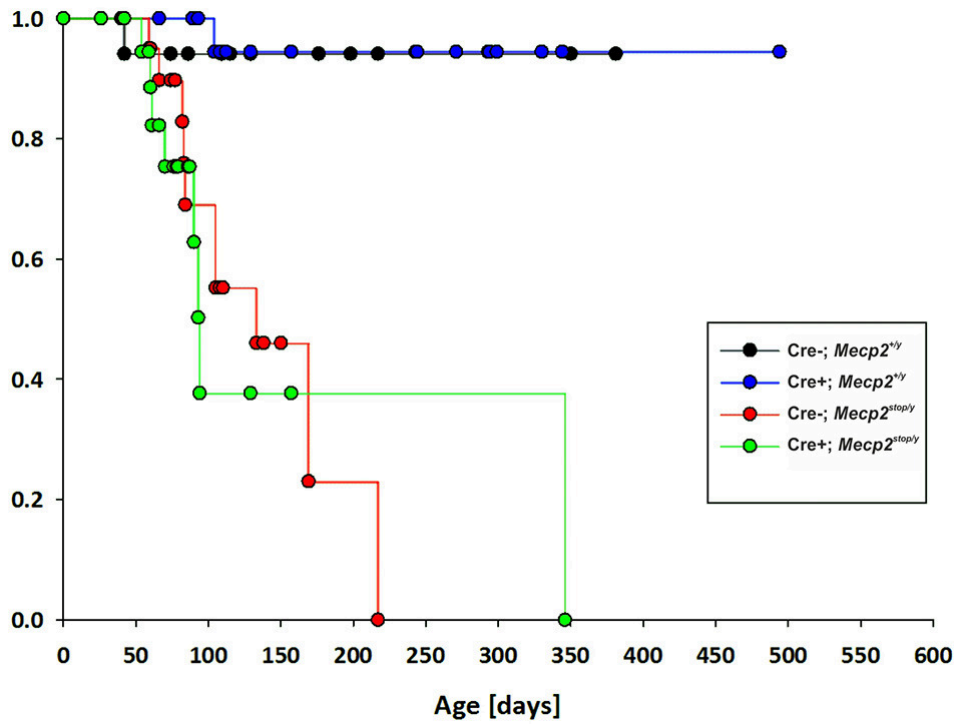
**Kaplan–Meier plot of survival for control mice (***GlyT2***-Cre; blue), MECP2-deficient ***Mecp2***^**stop/y**^ mice (red) and mice, in which MECP2 was expressed in ***GlyT2***-Cre positive neurons (Cre^**+**^; ***Mecp2***^**stop/y**^, green)**.

Although we observed a significant improvement of breathing at ages MECP2-deficient mice start to deteriorate, we did not find an improvement of the overall survival in *GlyT2*-Cre positive *Mecp2*^stop/y^ mice (Figure 3), indicating that the obvious improvement of breathing is not preventing a further progression of the disease.

## Discussion

Our data support the concept that a MECP2-deficiency in inhibitory neurons is a key factor in generation of the breathing phenotype in male MECP2-deficient mice. An early reintroduction of MECP2 in neurons, which express the glycine transporter 2, improves ventilation and eliminates prolonged periods of apnea, which both are the typical signs in male MECP2-deficient mice *in vivo* (Viemari et al., 2005; Chao et al., 2010; Wegener et al., 2014). This observation is in line with the results from a conditional *Mecp2* knock out in GABAergic neurons (Chao et al., 2010). Although the respiratory phenotype was observed to start rather late in the VIAAT-Cre mediated model—the data shown by Chao and colleagues were from postnatal week 32—the observed changes are typical for male MECP2-deficient mice (Chao et al., 2010).

In our hands, *Mecp2*^stop/y^ mice developed first signs of altered breathing after P40 and hypoventilation became significantly different to control mice around P60. When the stop-codon was deleted by the Cre-recombinase in *GlyT2*-Cre positive neurons the development of this typical change in breathing were alleviated (Figure 2) indicating that expression of MECP2 in hindbrain inhibitory neurons is sufficient for preventing the development of the respiratory phenotype. We assume that this rescue occurs in both type of inhibitory neurons in the respiratory network, since during embryonic development, *GlyT2*-Cre is expressed not only in glycinergic neurons (Gomeza et al., 2003b) but also in GABAergic neurons (Rahman et al., 2015). Additional experiments are required to distinguish between the role of GABAergic and glycinergic neurons.

However, reasonable evidence points toward disturbances in the GABAergic system. At first, 7 day old male MECP2-deficient mice show early changes in GABA_*A*_-receptor expression and postsynaptic GABA currents in the pre-Bötzinger Complex (Medrihan et al., 2008). Furthermore, substantial changes in GABAergic transmission in the Kölliker–Fuse region of the pons have been reported to contribute to the breathing phenotype in adult heterozygous females (Abdala et al., 2016). On the other hand, pharmacological improvements of breathing phenotype can also be attributed to an improvement of GABAergic transmission (Abdala et al., 2010; Voituron and Hilaire, 2011; Bittolo et al., 2016). Moreover, it is reasonable to assume that any change of GABAergic transmission that affects breathing is located in the hindbrain since neither the conditional *Mecp2* knock out in Parvalbumin-positive neurons (He et al., 2014; Ito-Ishida et al., 2015) nor in Somatostatin-positive neurons (Ito-Ishida et al., 2015) were able to mimic the breathing phenotype of the conditional VIAAT-dependent (Chao et al., 2010) or constitutional *Mecp2* knock out *in vivo* (Viemari et al., 2005).

We cannot exclude that the repression of MECP2 in *GlyT2*-Cre positive inhibitory neurons, which is supposed to start during embryonic development, is just delaying the onset of the respiratory phenotype by improving inhibitory neurotransmission without affecting the disturbance in glutamatergic neurons or glial cells. It was recently shown (Garg et al., 2015) that restoration of MECP2 in glutamatergic (*Vglut*-cre^+/−^) neurons significantly improved premature lethally of male MECP2-deficient mice (*Mecp*2^stop/y^). Although no information is giving regarding the respiratory phenotype in that publication, their data favor a role of glutamatergic neurons in the disease progression. Interestingly, restoration of MECP2 in astrocytes also improved both breathing and lifespan (Lioy et al., 2011). We can only speculate whether re-expression of MECP2 in astrocytes improved the breathing by effecting transport of the inhibitory neurotransmitters (Gomeza et al., 2003a; Szoke et al., 2006) and excitatory neurotransmitters (Schnell et al., 2011) or whether other factors unrelated to synaptic transmission in the respiratory network are important.

### Outlook

It has been suggested that hypoxia in younger MECP2-deficient mice and the resulting oxidative stress is an important factor for the progression and pathogenesis of Rett syndrome (De Felice et al., 2009, 2012). However, our data shine new light on this concept. Although breathing disturbances are strongly improved or at least delayed when MECP2 is preserved in inhibitory neurons, no prolongation of the life span was observed, suggesting that not the respiratory network failure but rather the loss of MECP2 in other brain region and especially in excitatory neurons is promoting the overall deterioration of MECP2-deficent mice.

## Author contributions

SH, MN designed experiments. SH, GM, JD, MN conducted experiments. MA, MN, SH analyzed data. SH wrote the paper.

### Conflict of interest statement

The authors declare that the research was conducted in the absence of any commercial or financial relationships that could be construed as a potential conflict of interest.
